# Colloidal Silver Induces Cytoskeleton Reorganization and E-Cadherin Recruitment at Cell-Cell Contacts in HaCaT Cells

**DOI:** 10.3390/ph12020072

**Published:** 2019-05-15

**Authors:** Elena Montano, Maria Vivo, Andrea Maria Guarino, Orsola di Martino, Blanda Di Luccia, Viola Calabrò, Sergio Caserta, Alessandra Pollice

**Affiliations:** 1 Dipartimento di Biologia, Università degli Studi di Napoli Federico II, Via Cintia 21, 80126 Napoli, Italy; elenamontano26@gmail.com (E.M.); maria.vivo@unina.it (M.V.); am.guarino91@gmail.com (A.M.G.); ors.dimartino@gmail.com (O.d.M.); blanda.diluccia@gmail.com (B.D.L.); vcalabro@unina.it (V.C.); 2 Dipartimento di Ingegneria Chimica dei Materiali e della Produzione Industriale (DICMAPI) Università degli Studi Napoli Federico II, P.le Tecchio, 80, 80125 Napoli, Italy

**Keywords:** colloidal silver, wound healing, E-cadherin, keratinocytes, nanoparticles, skin

## Abstract

Up until the first half of the 20th century, silver found significant employment in medical applications, particularly in the healing of open wounds, thanks to its antibacterial and antifungal properties. Wound repair is a complex and dynamic biological process regulated by several pathways that cooperate to restore tissue integrity and homeostasis. To facilitate healing, injuries need to be promptly treated. Recently, the interest in alternatives to antibiotics has been raised given the widespread phenomenon of antibiotic resistance. Among these alternatives, the use of silver appears to be a valid option, so a resurgence in its use has been recently observed. In particular, in contrast to ionic silver, colloidal silver, a suspension of metallic silver particles, shows antibacterial activity displaying less or no toxicity. However, the human health risks associated with exposure to silver nanoparticles (NP) appear to be conflicted, and some studies have suggested that it could be toxic in different cellular contexts. These potentially harmful effects of silver NP depend on various parameters including NP size, which commonly range from 1 to 100 nm. In this study, we analyzed the effect of a colloidal silver preparation composed of very small and homogeneous nanoparticles of 0.62 nm size, smaller than those previously tested. We found no adverse effect on the cell proliferation of HaCaT cells, even at high NP concentration. Time-lapse microscopy and indirect immunofluorescence experiments demonstrated that this preparation of colloidal silver strongly increased cell migration, re-modeled the cytoskeleton, and caused recruitment of E-cadherin at cell-cell junctions of human cultured keratinocytes.

## 1. Introduction

The use of silver in therapeutic applications has very ancient origins due to its broad and highly effective antibacterial activity [1,2]. However, the scientific debate on its mechanism of action is still an open field. Some authors maintain that the bactericidal activity could be linked to the release of silver ions [3,4,5,6,7,8] and their interaction with several bacterial components such as peptidoglycan, the cell membrane as well as bacterial proteins/enzymes involved in vital processes [8,9,10,11]. In contrast, other authors assert that the effect of silver could be due to a physical effect toward the cell membrane with consequent penetration of silver inside the cytoplasm and interference with cellular components [10]. More recently, another important effect exerted by silver preparations was described, which regarded the inhibition of bacterial biofilm formation [12,13]. Biofilms (aggregates of bacteria embedded in an extracellular matrix) allow bacterial growth in a protective environment, reducing antibiotic susceptibility and favoring escape from the immune response.

In addition to these functions, it has been demonstrated that silver has anti-inflammatory effects and improves the healing of wounds through the modulation of fibrogenic cytokines [12,13,14,15,16] and a decrease in lymphocyte infiltration [17,18]. Thus, silver has found wide applications in preventing further injury and bacterial invasion of wounds, therefore improving the efficient recovery of damaged tissues.

All of these characteristics have caused a widespread use of silver, both in medicine and daily life. Nowadays, medical devices (dressing for wounds, surgical catheters, stitches, bone cement) as well as cosmetics, cosmeceuticals, and tessils, have a silver component [13,16,19,20]. Nevertheless, although it is considered relatively non-toxic to mammals, chronic exposure to Ag+ ions determine the formation of insoluble precipitates in the dermis and the cornea/conjunctiva, causing the so-called argyria or argyrosis syndromes, blue coloration of the skin, and mucous tissues [3,4].

It is essential to underline that most of the toxicity of silver is because ionic silver (Ag+) is exceptionally reactive toward molecules and cellular structures [21]. Therefore, especially during the last few years, ionic silver usage has been superseded by colloidal silver, i.e., as a suspension of microscopic metallic nanoparticles (NP up to 100 nm in diameter), presenting lower toxicity with respect to their metallic counterpart [3,4].

Notions about colloidal silver safety and biocompatibility have appeared to sometimes be contradictory in the literature. Several studies have confirmed silver NP as clinically safe [22], and dressings based on silver NPs have been declared safe for patients [20,23,24]. However, some studies have shown that NPs are cytotoxic for several different cell lines, mostly by inducing an increase of ROS production, a decrease in mitochondrial function, DNA damage, and apoptosis [25,26,27,28,29,30]. In other cases, a decrease in cell proliferation without DNA damage has been reported [31]. In contrast, studies performed in human fibroblasts confirmed that AgNPs could alter mitochondrial functionality without leading to cell death [16] and one study identified a relationship between NP size and inhibitory effects on mitochondria [32].

It has to be noted that silver nanoparticles can be very heterogeneous and such heterogeneity could probably in part explain differences present in the literature. Several methods to produce silver NP have been developed. Preparations of colloidal silver commercially available (see http://www.silver-colloids.com/) can differ for NP size, stability (Zeta potential), concentration, and different percentages of ionic silver either due to the efficiency or synthesis methods. Parameters that are influenced by the synthesis method include the mean NP diameter and size, size distribution, shape, stability, the inclusion of ligand shells, and capping agents [21].

Considering the widespread use of silver NP and the growing interest in its use due to its versatility, we analyzed the biological properties of a colloidal silver preparation with silver NP of 0.62 nm in size, smaller than those ever described and presenting an extremely low percentage of ionic silver [33,34,35]. We first evaluated the antimicrobial activity with results similar to what has already been published [36,37,38,39] with the data not shown. Then, we assessed the effect on a model of human skin, HaCaT human keratinocytes. We observed no toxicity by the MTT assay, growth curve analysis, absence of stress granules, and strong efficacy in promoting wound healing in vitro. Interestingly, colloidal silver induced an evident cytoskeleton reorganization accompanied by an increase in cell–cell junctions underlined by E-cadherin recruitment.

## 2. Results

### 2.1. Effect of Colloidal Silver on HaCaT Cells

**Figure 1:**
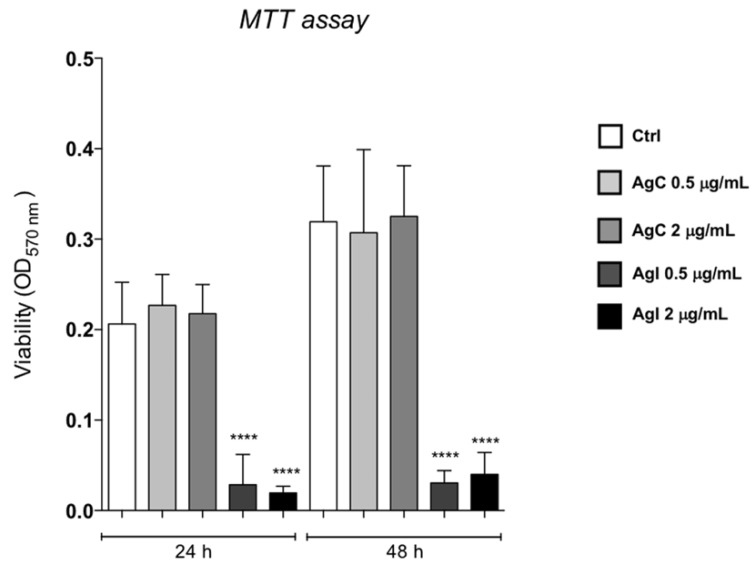
Effects of AgC on HaCaT cell viability. MTT assay of HaCaT cells incubated for 24 or 48 h with colloidal (grey bars) or ionic silver (dark bars) at 0.5 or 2 μg/mL. Data are expressed as absorbance at 570 nm and presented as mean ± SE of three independent experiments, each done in sestuplicate. Analysis of variance was performed by two-way Anova followed by the Bonferroni post-test. **** P < 0.0001 when compared with the control.

Initial experiments investigated the potential toxicity of colloidal silver on human immortalized HaCaT keratinocytes. For this purpose, HaCaT cells were grown in the presence of colloidal or ionic silver at 0.5 or 2 μg/mL for 24 and 48 h and cell viability was analyzed by the MTT assay as described in [40]. Figure 1 shows that colloidal silver did not exert any toxic effect, while ionic silver caused a dramatic reduction of cell viability at both concentrations.

**Figure 2:**
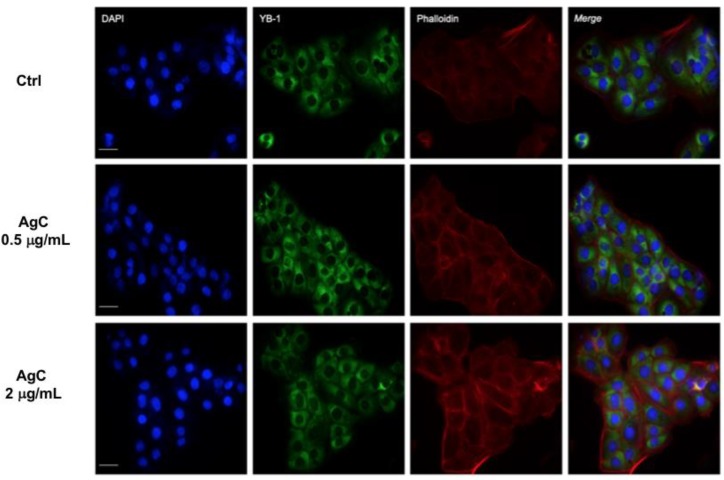
Effect of AgC on stress granules formation. HaCaT cells were seeded on a coverslip and treated (or not) with AgC at 0.5 or 2 μg/mL for 24 h. Cells were then fixed and analyzed by TRITC-conjugated phalloidin staining (red) or indirect IF with the anti-YB1 antibody (green). Nuclei were stained with DAPI. Scale bar, 7 μM. Images were acquired using a Nikon TE Eclipse 2000.

Importantly, cell viability increased between 24 and 48 h even when colloidal silver was added at the higher concentrations of 2 μg/mL, thus suggesting no toxicity under these conditions. We therefore addressed whether colloidal silver could function as a stressor agent by analyzing the stress granules formation (SG). SG are aggregates of proteins and RNA that form when cells are subjected to different kind of stresses to protect cellular structures from harmful conditions. To monitor the formation of these aggregates, we looked at the YB-1 protein as a specific marker of SG [41]. Furthermore, it has to be noted that YB-1 typically translocates to the nucleus following genotoxic stress [42], therefore, it can also be used to monitor harmful insult to the cells. HaCaT cells were grown in the presence of colloidal silver at 0.5 and 2 μg/mL for 24 h, fixed, and analyzed by indirect IF with the anti-YB1 antibody. Figure 2 clearly shows that colloidal silver induced neither stress granules formation nor YB-1 nuclear translocation. Cells were also stained with TRITC-conjugated phalloidin to visualize the actin cytoskeleton. The experiment showed that colloidal silver treatment induced actin cytoskeleton rearrangement at the cellular periphery. In particular, we observed increased f-actin polymerization both at the cell–cell and cell–substratum adhesions (Figure 2, red panels).

### 2.2. Effect of Colloidal Silver on Wound Healing

**Figure 3:**
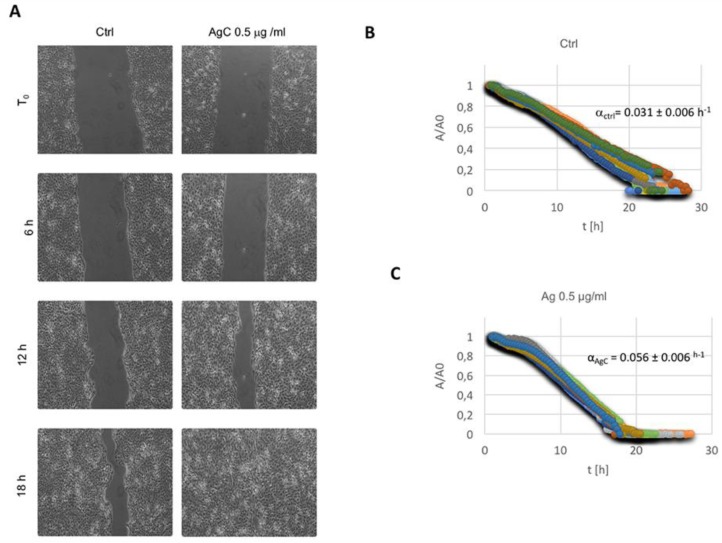
Effect of AgC on wound closure dynamics. (**A**) Representative phase contrast microscopy images of cells incubated (or not) with AgC at different time points showing the wound closure process over time. (**B**,**C**) The wound closure process was quantified by measuring the reduction of the wound area (**A**) over time, as described in the Materials and Methods Section. Evolution of the wound area A, normalized to the value A0 (A at time 0), is reported for the control (**B**) and AgC treated (**C**) cells by selecting random fields along the wound for each experiment. The linear range of each data series was fit in order to measure the wound closure velocity α. Values of α for the control and treated cells are indicated on each graph as the mean from three independent experiments analyzed in triplicate. Standard error of the mean was calculated to account for reproducibility, and the t-test was calculated to verify the statistical significance of the differences with respect to the control samples.

Based on these data showing a reorganization of the actin cytoskeleton, we decided to analyze the effect of colloidal silver on cell migration, a process where massive cytoskeleton dynamics take place. Wound healing activity was evaluated in vitro by automated time-lapse microscopy [43]. Details on the technique are reported in the Materials and Methods Section. Typical images of the wound as a function of time are presented in Figure 3A, where samples in the absence and/or presence of AgC 0.5 μg/mL are compared.

Images at the same time points show better closure of the wound for the treated sample with respect to the control. This result was systematic, as visible from Figure 3B,C, where the evolution of the wound area, normalized with respect to its initial value (A/A0), is reported for the control and treated sample, respectively. Raw data reported in the two diagrams for each independent field of view showed excellent reproducibility. We calculated the values of the wound closure velocity (α) from each data series, as reported in the Materials and Methods Section. Interestingly, the αAgC/αcontr (that is a measure of the relative effect of the treatment in our conditions) was 1.80, indicating that the wound closure velocity roughly doubled in the presence of AgC 0.5 μg/mL.

**Figure 4:**
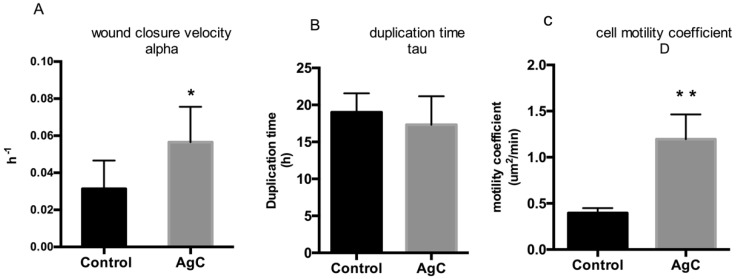
Effect of AgC on wound closure dynamics. Values of α (wound closure velocity) (**A**), τ duplication time) (**B**), and D (cell motility coefficient) (**C**) of the control and AgC treated cells are reported. Values of D were calculated according to the Fisher–Kolmogoroff equation from values of α and τ (see Materials and Methods). Data are expressed as the mean of at least three independent experiments. SEM is reported as error bars, statistical significance was assessed by the paired two-tailed t-test (* P = 0.04; ** P = 0.007).

In Figure 4B, the duplication time (τ) of HaCaT cells grown in the presence and/or absence of 0.5 μg/mL AgC is reported. Intriguingly, cell duplication was not significantly altered in the presence of colloidal silver, while the cell motility coefficient calculated from the Fisher–Kolmogoroff equation appeared to be drastically and statistically significantly increased (Figure 4C). Overall, these results indicate that under these experimental conditions, colloidal silver affected cell motility more than cell proliferation.

### 2.3. Effect of Colloidal Silver on Cell-Cell Contacts and Cell Morphology

**Figure 5:**
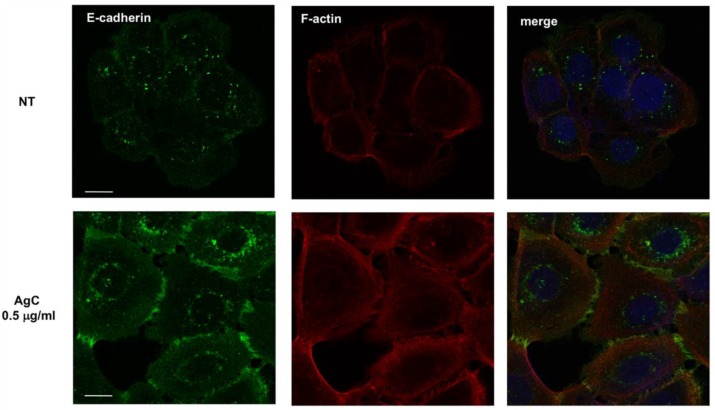
E-cadherin localization in HaCaT cells upon AgC treatment. Cells were allowed to adhere onto coverslips for 24 h and then treated with 0.5 μg/mL AgC for 8 h. Cells were then fixed and subjected to IF with an anti-E-cadherin antibody followed by TRITC-conjugated phalloidin to visualize the actin cytoskeleton. Representative images of E-cadherin subcellular localization and phalloidin are shown. Merged images also show DAPI staining to visualize the nuclei. Images were taken with a Zeiss confocal laser-scanning microscope Axio Observer (scale bar, 15 μM). A ×40 objective was used and image analysis was performed using ImageJ.

Regulation of cell shape and motility is governed in large by the cytoskeleton, of which actin filaments are the major components. Cytoskeletal elements influence the formation of cell–cell and cell–substrate adhesions that play a fundamental role in both cell morphology and migration which requires the continuous assembly and disassembly of cellular adhesions. To further characterize the effects of AgC on cell shape, the cytoskeleton and cell–cell contacts were examined by fluorescence microscopy by both phalloidin staining and E-cadherin immunofluorescence. Cells were allowed to adhere onto coverslips overnight, treated with 0.5 μg/mL AgC for 8 h, fixed, and subjected to IF with anti-E-cadherin, followed by TRITC-conjugated phalloidin incubation to visualize the actin cytoskeleton and DAPI to stain the nuclei. As previously observed, cells displayed a dense meshwork of actin filaments around the cell periphery. Interestingly, the experiment showed that AgC treatment caused significant recruitment of E-cadherin at cell–cell junctions, which were apparent within 8 h of treatment (Figure 5). Similar results were obtained when cells were incubated with AgC for 24 h.

## 3. Discussion

In this work, we evaluated the use of a colloidal silver solution in the cell viability and cell migration of immortalized human keratinocytes. Although the use of silver in medicine dates back to ancient times, its use became less frequent upon the widespread use of antibiotics. The appearance in recent decades of the increasingly dangerous phenomenon of antibiotic resistance has brought to the forefront the use of silver as a valid non-toxic alternative. Our experiments showed that no adverse effect could be observed in the HaCaT cells by both the MTT assay and stress granules formation when we treated keratinocytes at even rather high concentrations (2 μg/mL) of AgC. Interestingly, upon treatment, cells tended to reorganize the cytoskeleton as indicated by the observation of phalloidin-stained F-actin. Since tissue healing is linked to a profound reorganization of the cytoskeleton, we evaluated the activity of silver in wound healing by following the wound healing process using time-lapse video-microscopy. The algorithm we used allowed for the quantitative analysis of the dynamics in the reduction of the cutting area. Our data indicated that in the presence of silver, the wound closure speed increased and cells incubated with colloidal silver “healed the damaged area” in less time than the controls. This result was quantified and interpreted according to the Fisher–Kolmogoroff model [43], which allows the process of re-closing the cut mathematically to be described. A novel data analysis approach was used to identify the relative role of cell motility and proliferation on wound healing through simple calculations. We found that AgC increased the mechanism of cell migration rather than increasing the cell proliferation levels. Cell motility is primarily governed by the cytoskeleton, which influences both the cell–substrate and cell–cell contacts. Our results were supported by immunofluorescence experiments, where HaCaT cells presented an overall increased E-cadherin signal and its re-localization to the cellular periphery and the cell–cell junctions. It is interesting to note that it has been reported that the intercellular junctions marked by E-cadherin allow cells to communicate [44] and to move in a highly coordinated way [45]. The increase in cell–cell junctions through strong E-cadherin-mediated contacts at the upper edge, in the lateral regions, and within the moving cell group characterizes the collective cell migration [44,46,47,48]. This aspect is particularly interesting, since collective cellular migration would be the basis of the movement and proliferation of epidermal keratinocytes located on the edge of the wound following skin lesions [49]. It is interesting to note that cell movements are characterized by cytoskeletal reorganization through the formation of adherent junctions [49]. The adherent junctions, favored by the recruitment of E-cadherin on the cell surface, allow for anchorage to the actin cytoskeleton [50] through their binding with both α- and β-catenins. The increase of the actin polymerization induced by colloidal silver and the E-cadherin relocation to the cell–cell junctions both indicate that colloidal silver is broadly involved in the cytoskeletal reorganization occurring during migration. In agreement, it has been observed that silver nanoparticles are able to increase connexin 43-mediated gap junctional intercellular communication in HaCaT cells [51]. Interestingly, this occurs through ROS production and activation of the MAPK pathway. Furthermore, in an in vitro model of the human gut epithelium, exposure to AgNP caused changes in cellular permeability and a dysregulation in the expression of components of tight junctions and desmosomes without affecting E-cadherin; however, it has to be noted that such experiments have been performed with NP of 10 nm in size, about 18x bigger than the ones used in the present study [52].

## 4. Conclusions

Altogether, the presented data indicate that colloidal silver could improve the healing process by modulating the reorganization of the cytoskeleton and thus cell motility. Further analysis is needed to clarify both the mechanism of action and the molecular pathways involved. However, the collected data encourage further investigation of AgC in tissue healing. In this regard, therapeutic agents including steroids, glucocorticoids, non-steroidal anti-inflammatory drugs, and chemotherapy are associated with several side effects that, by interfering with cell movement in the wound, slow down the repair process [53]. In this scenario, silver could have a positive effect on wound closure by acting on different levels and with different mechanisms compared to the generally used drugs. Last but not least, it has to be underlined that thanks to its antimicrobial properties, silver could be a valid alternative for the treatment of infections also caused by multi-drug-resistant bacteria (MDRB) [54], which do not respond to standard pharmacological therapies and for which it is challenging to develop efficient treatments.

## 5. Materials and Methods

### 5.1. Cell Culture and Reagents

HaCaT, spontaneously immortalized keratinocytes from adult skin, were purchased from Cell Line Service (CLS, Eppelheim, Germany) and cultured in Dulbecco’s Modified Eagle’s Medium (DMEM, Sigma Chemical Co., St. Louis, MO, USA) supplemented with 10% fetal bovine serum (FBS, Hyclone Laboratories, Inc., Logan, UT, USA), 1% L-glutamine, and 1% penicillin-streptomycin (ICN Biomedicals, Inc., Aurora, OH, USA) at 37 °C in a humidified atmosphere of 5% CO_2_ [55,56]. Depending on the type of experiment, HaCaT cells were seeded at different densities and on different culture dishes. The cells were treated with a colloidal silver preparation containing 79.5% silver nanoparticles of 0.62 nm in size, obtained from Santé Naturels SNC (Civitanova Marche, Macerata, Italy) at the concentration of 20 ppm (20 μg/mL). Detailed information regarding the characteristics of the colloidal silver solution are visible at the following official site: http://www.silver-colloids.com/Click comparison table; European Products Reports). Further information regarding the size of the nanoparticles and the Zeta potential (index of stability) are presented in the Supplementary Materials, S1, S2). For each experiment, the stock solution was diluted in culture medium. Ionic silver was obtained by dissolving 3.15 mg AgNO_3_ in 100 mL H_2_O to obtain a final stock solution where the ionic silver was 20 μg/mL.

### 5.2. Cell Viability (MTT Assay) and Cell Proliferation Assays

The effect of ionic and colloidal silver on cell viability was evaluated by measuring the reduction of 3-(4,5-dimethylthiazol-2) 2,5-diphenyltetrazolium bromide (MTT) to formazan by mitochondrial dehydrogenase [57,58]. Briefly, 9 × 10^3^ cells were seeded on 96-well plates and exposed to increasing concentrations of either 0.5 or 2 μg/mL colloidal silver for 24 and 48 h. MTT/PBS solution (0.5 mg/mL) was then added to the wells and incubated for 3 h at 37 °C in a humidified atmosphere. The reaction was stopped by the removal of the supernatant, followed by dissolving the formazan product in acidic isopropanol. Optical density was measured with an ELISA reader (Bio-Rad) using a 570 nm filter using an iMark microplate reader (Bio-Rad, Hercules, CA, USA). Each experiment was performed in quadruplicate, in three independent experiments. The cell viability was calculated as CV (%) = (Absorbance of test sample/Absorbance of control) × 100.

For cell proliferation analysis, cells were incubated with AgC and the number of cells in each experimental point was counted with a Scepter-Millipore counter (Handheld Automated Cell Counter) as described in [59]. Growth curves were generated and the cell population doubling time (τ) was estimated by fitting a typical logistic growth [60] (Equation (1)) where τ was the only adjustable parameter.

(1) n(t)=n0∗2(tτ)

### 5.3. In Vitro Wound Scratch Assay

To test the effect of colloidal silver on the wound closure phenomenon, HaCaT cells were seeded in 12-well plates at a density of 4.5 × 10^5^ cells/well. The day after plating, once they had reached 90–100% confluence, cells were starved for 6 h in serum-free DMEM to completely inhibit cell proliferation. The confluent monolayer was scraped with sterile P200 pipette tips and washed twice with PBS to remove detached cells and debris. The scratched monolayers were treated with colloidal silver (0.5 μg/mL) diluted in culture medium and plates were incubated as described. Wound closure was monitored in the different samples by automated time-lapse microscopy (TLM) using an inverted microscope (Zeiss Axiovert 200, Carl Zeiss, Germany) inserted into an incubator with constant T (37 °C), humidity (100% Hr), and CO_2_ (5%). Different fields of view in each cell dish were acquired by phase contrast microscopy using a CCD video camera (Hamamatsu Orca AG, Japan) at regular intervals (15 minutes) for about 18 h using a long working distance 5X objective in phase contrast (CP Achromat Ph1). Images were analyzed as described in [43]. The wound closure dynamics were quantified by using a homemade automated image analysis software, which allowed us to measure the size of the wound area for each time point. In a typical experiment, for each field of view, the cell nude area (A) was measured for each time step, normalized with respect to the value of the wound area at time 0 (A0), and plotted as a function of time. After an initial Lag time (tL), A/A_0_ was found to decrease with a constant velocity; the slope of the linear range of the A/A_0_ vs. t curve (α) can be considered as a measure of the wound closure velocity. Details on the calculation of t_L_ and α are reported elsewhere (see Figure 9b in [61]. For each experimental condition (control and treated), at least four independent fields were analyzed out of three independent wells for each experiment. Each experiment was done in triplicate and statistical analysis performed by calculating the standard error of the mean (reported as error bar) and calculating statistical significance by testing the null hypothesis (t-test).

### 5.4. Wound Healing Data Analysis

The wound healing process can be modeled according to the Fisher–Kolmogoroff equation (Equation (2)), which is a diffusion-reaction equation based on a transport phenomenon approach [62,63]. This model mathematically describes the evolution of the cell density profile u, which depends on the time and distance x, measured from the wound edge, i.e., along the wound closure direction, that is, the horizontal direction in the images reported in this work. Two different phenomena contribute to wound closure, i.e., cell motility and proliferation, which are both involved in the spatial spreading of the cells in the wound region, and are both summed at the right side of Equation (2).

(2) $$\frac{\partial u}{\partial t}=D\frac{\partial^{2}u}{\partial x^{2}}+\mathrm{ku}\left(1-\frac{u}{\hat{u}}\right)$$

Cell motility is modeled as a Fickian diffusion, according to the assumption of a persistent random motion of the cells [64,65,66] and depends on a random motility coefficient, that is, analogous of a diffusion coefficient (D, in Equation (2)). Cell proliferation can be modeled (last term in Equation (2)) as a logistic growth, where the growth velocity is reduced as the cell density approaches confluence, which is measured by the maximum cell density $\hat{u}$. k is a growth kinetic constant, which as a first approximation can be estimated as the reciprocal of the cell doubling time ($k=ln_{2}(\tau$) [62,67]. This model predicts that after a short transient phase, the wound edges propagate at a constant speed in the direction x, perpendicular to the wound edge, reducing the size of the wound area. The speed of propagation of each wound edge v is related to the values of the random motility coefficient and of the cell doubling time (Equation (3)) [62,67].

(3) $$v=\sqrt{\frac{4Dln\left(2\right)}{\tau }}$$

The concurring role of cell motility and proliferation can be estimated by the simple calculation of the Thiele modulus $\phi =\frac{b}{2}\sqrt{\frac{K}{D}}$ [62], where b is the initial wound size, i.e., the distance of the two edges of the wound at time 0. Given the measure of the wound closure velocity α from the analysis of the time lapse experiments, it was easy to calculate the velocity of the propagation of the wound edges that is related to α by a simple geometrical relationship v = α* b/2, where b is the initial size of the wound, with high precision. The cell doubling time τ was independently calculated from cell proliferation assays (Equation (3)). Cell random motility coefficient D was finally calculated from reverse Equation (3).

(4) $$\left(D=\tau \frac{v^{2}}{4ln\left(2\right)}\right)$$

It is worth mentioning that the direct measurement of the cell random coefficient is a non-trivial task that requires time consuming tracking of cell motion over time [43]. The advantage of the approach used here is the possibility of estimating such a relevant parameter from the simple analysis of wound healing experiments, and trivial algebraic calculations based on advanced models.

### 5.5. Immunofluorescence

IF assays were performed as previously described in [68,69]. Briefly, treated and control HaCaT cells were seeded on glass coverslips at a density of 1.5 × 10^5^ cells/well in 24-well dishes, fixed with 3.7% PFA, and permeabilized with 0.5% Triton X-100. After blocking with 3% BSA, cells were incubated with primary antibody (E-cadherin and YB-1) for 1 h at RT followed by incubation with Alexa-Fluor conjugated secondary antibodies for 1 h in the dark. To visualize the actin cytoskeleton, cells were stained with TRITC-conjugated phalloidin. The cells were counterstained with DAPI for the visualization of the nucleus. Images were taken with a Zeiss confocal laser-scanning microscope Axio Observer. A x40 objective was used and image analysis was performed using ImageJ. All images were taken with the same setting. Image processing and analysis were performed with Fiji (ImageJ version 2.0) software. The stress granules formation experiment was performed as described in [70] and images were acquired using a Nikon TE Eclipse 2000. Antibodies of anti-YB-1 (12148 Abcam, Cambridge, UK), anti-E-Cadherin (610181 BD Transduction Laboratories™, MA, USA), Alexa Fluor 488 anti-rabbit and anti-mouse (Thermo-Fisher Scientific, Waltham, MA, USA), and DAPI (Sigma-Aldrich, Saint Louis, MO, USA) were used.

### 5.6. Statistical Analysis

All data are expressed as the means of independent experiments (biological replicates) ± standard errors (SE). Analysis of variance was performed by a two-way ANOVA followed by the Bonferroni post-test using Graph-Pad Prism (Graph-Pad Software), or by the Student’s T-test as previously described in [59].

## Supplementary Materials

The following are available online at https://www.mdpi.com/1424-8247/12/2/72/s1.
