# Supplementary material for: Colloidal Silver Induces Cytoskeleton Reorganization and E-Cadherin Recruitment at Cell-Cell Contacts in HaCaT Cells
Source: Pharmaceuticals (Basel). 2019 May 15;12(2):72. doi: 10.3390/ph12020072 (PMC6631624; doi:10.3390/ph12020072)

NANO ZS

Size Distribution Report by Volume

Sample Details

Sample Name: ms-120207-2-avg

SOP Name: CSL-00.sop

General Notes: Average result created from record number(s): 260 261 262 263 264 265 266 267 268 269

**File Name:**	CSL-2012-size.dts	**Dispersant Name:**	Water
**Record Number:**	270	**Dispersant RI:**	1.330
**Material RI:**	1.25	**Viscosity (cP):**	0.8872
**Material Absorbtion:**	0.01	**Measurement Date and Time:**	Friday, February 10, 2012 1:...

System

**Temperature (°C):**	25.0	**Duration Used (s):**	70
**Count Rate (kcps):**	190.5	**Measurement Position (mm):**	4.65
**Cell Description:**	Disposable sizing cuvette	**Attenuator:**	9

Results

Polydispersity Index: 0.827

	Diam. (nm)	% Volume	Width (nm)
**Peak 1:**	0.62	99.8	0.1
**Peak 2:**	1.80	0.1	0.3
**Peak 3:**	4.72	0.1	1.7

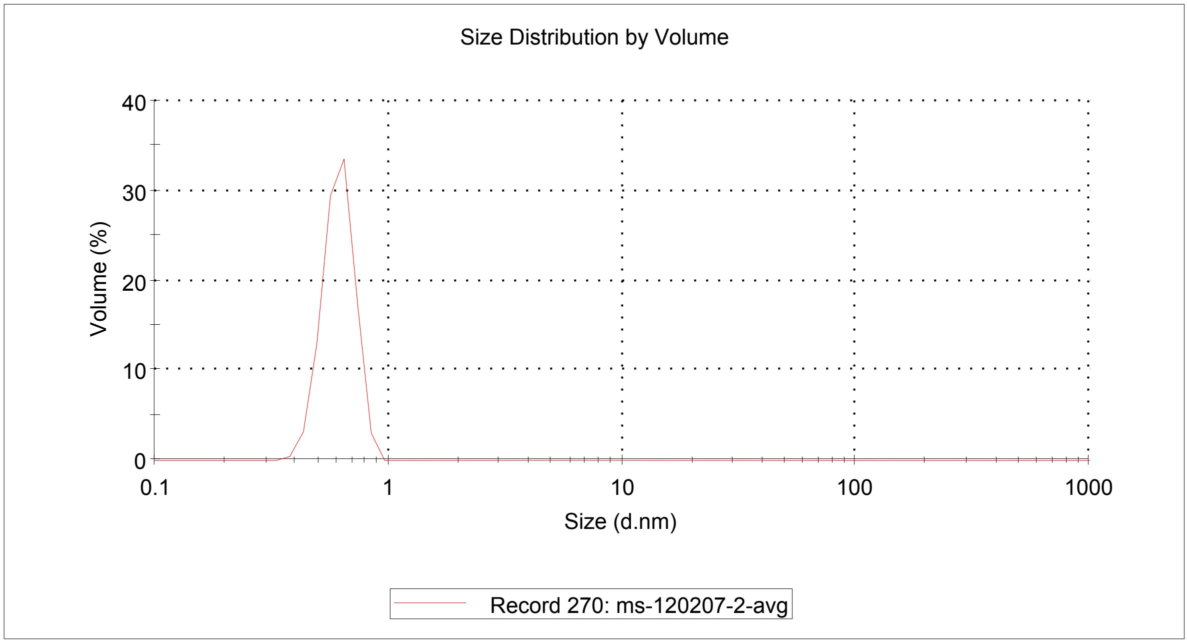

Colloidal Science Laboratory, Inc. **Westampton, NJ 609-267-2065**

Zeta Potential Report

v2.2

Malvern Instruments Ltd - © Copyright 2008

Sample Details

**Sample Name:**	nano cristal silver 1
**SOP Name:**	mansettings.nano
**General Notes:**	

**File Name:**	Santè Naturels.dts	**Dispersant Name:**	Water
**Record Number:**	7	**Dispersant RI:**	1,330
**Date and Time:**	lunedì 12 novembre 2012 17:2...	**Viscosity (cP):**	0,8872
		**Dispersant Dielectric Constant:**	78,5

System

**Temperature (°C):**	25,0	**Zeta Runs:**	12
**Count Rate (kcps):**	66,6	**Measurement Position (mm):**	2,00
**Cell Description:**	Clear disposable zeta cell	**Attenuator:**	11

Results

			**Mean (mV)**	**Area (%)**	**Width (mV)**
**Zeta Potential (mV):**	-33,5	**Peak 1:**	-33,5	100,0	6,43
**Zeta Deviation (mV):**	6,43	**Peak 2:**	0,00	0,0	0,00
**Conductivity (mS/cm):**	0,0268	**Peak 3:**	0,00	0,0	0,00

Result quality Good

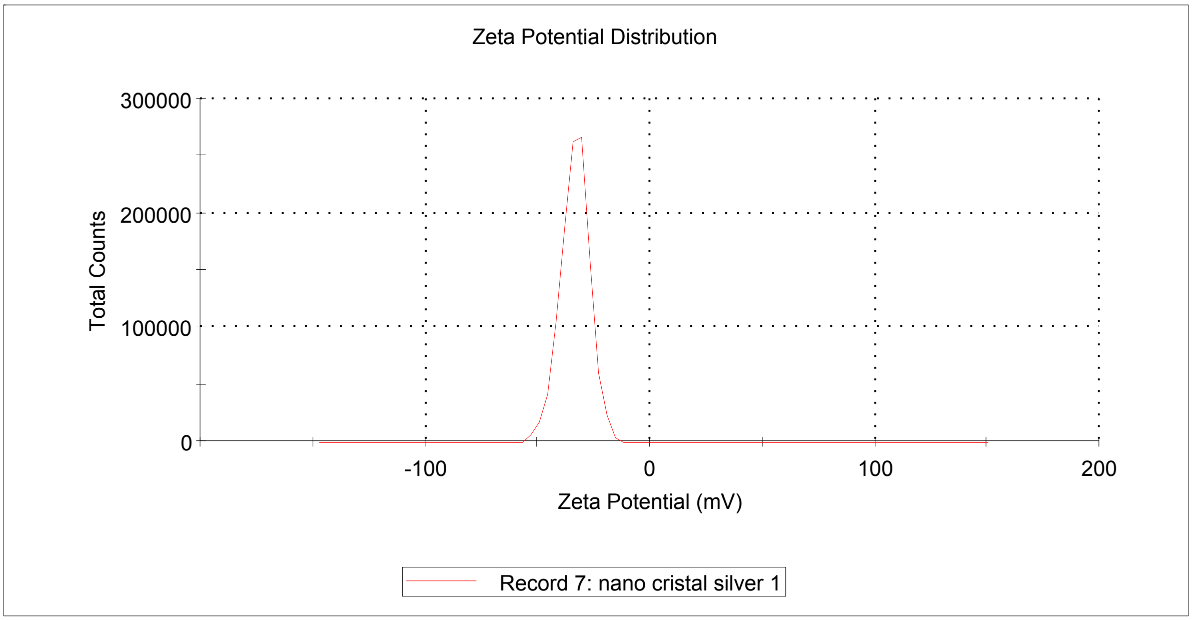

Supplement: Supplementary file 1 [file pharmaceuticals-12-00072-s001.pdf]
